# A novel *in vivo* method to quantify slit diaphragm protein abundance in murine proteinuric kidney disease

**DOI:** 10.1371/journal.pone.0179217

**Published:** 2017-06-12

**Authors:** Raphael Haase, Sebastian Alexander Potthoff, Catherine Meyer-Schwesinger, Clara Frosch, Thorsten Wiech, Ulf Panzer, Eva Königshausen, Johannes Stegbauer, Lorenz Sellin, Lars Christian Rump, Ivo Quack, Magdalena Woznowski

**Affiliations:** 1 Department of Nephrology, Medical Faculty, Heinrich-Heine University, Düsseldorf, Germany; 2 III. Medical Clinic University Hospital Eppendorf, Hamburg, Germany; 3 Institute of Pathology, University Hospital Eppendorf, Hamburg, Germany; University of Houston, UNITED STATES

## Abstract

Injury of the glomerular filter causes proteinuria by disrupting the sensitive interplay of the glomerular protein network. To date, studies of the expression and trafficking of glomerular proteins have been mostly limited to *in vitro* or histologic studies. Here, we report a novel *in vivo* biotinylation assay that allows the quantification of surface expression of glomerular proteins in mice. Kidneys were perfused *in situ* with biotin before harvest. Afterwards glomeruli were isolated and lyzed. The protein of interest was separated by immunoprecipitation and the amount of surface-expressed protein was quantified by Western blot analysis with streptavidin staining. As proof-of-concept, we examined the presence of nephrin in the slit diaphragm in two well-established murine models of proteinuric kidney disease: nephrotoxic nephritis and adriamycin nephropathy. In proteinuric animals, significantly less nephrin was detected in the slit diaphragm. When proteinuria decreased once again during the course of disease, the amount of surface nephrin returned to the baseline. Our present results suggest that our assay is a valuable tool to study the glomerular filter in proteinuric kidney diseases. Note that the assay is not limited to proteins expressed in the slit diaphragm, and all surface proteins that are accessible to biotin perfusion and immunoprecipitation qualify for this analysis.

## Introduction

Injury to a single layer of the glomerular filter is sufficient to induce albuminuria. If podocytes and the slit diaphragm are involved, albuminuria easily reaches nephrotic ranges. Albuminuria is a main driver of the progression of kidney disease [1] and is associated with a markedly increased cardiovascular risk [2]. Several studies have suggested that altered trafficking of slit diaphragm proteins causes filter leakage. We previously demonstrated that nephrin, the backbone of the slit diaphragm, undergoes β-arrestin2-dependent endocytosis in healthy and diseased states [3, 4]. Meanwhile the concept of nephrin endocytosis has been confirmed and extended by other groups (reviewed in [5–7]). Newly identified regulatory mechanisms of nephrin endocytosis involve planar cell polarity pathways, e.g. Wnt5a and Vangl2 [8, 9]. CIN85/RukL controls nephrin turnover by ubiquitination [10, 11]. Some studies demonstrated dynamin- and clathrin-dependent uptake whilst others showed that raft-dependent endocytosis might be an alternative pathway [4, 12, 13]. Recent work suggests that reduction of endocytotic processes is also detrimental to the podocyte [14].

Studies of podocyte protein trafficking have been hampered by the lack of a suitable *in vivo* model of the glomerular filter. To elucidate the complex interplay of the filter structures mainly two different techniques have been used so far. First, the classical immunofluorescence labeling approach using antibodies that recognize epitopes in the extracellular domains of the receptors being studied [15]. Second, covalent modification of cell surface receptors with biotin as a valuable technique for monitoring surface protein trafficking. Biotinylation enables labeling of the cell surface protein at a particular time point, but instead of using a specific antibody, all surface proteins are tagged with biotin. The protein of interest is then separated by immunoprecipitation followed by SDS-PAGE and Western blotting. The biotinylated fraction can be visualized by staining with streptavidin and quantified by densitometry. So far biotinylation has been limited to *in vitro* experiments. Satoh et al. recently described a method in which glomeruli are extracted first and are then biotinylated in vitro [16]. However, it is known that the complex architecture of the glomerular filter is altered by mechanical stress. We learned from our previous work that especially the molecular composition of the slit diaphragm changes rapidly. Based on these observations we developed an *in vivo* biotinylation assay because we were convinced that biotinylation of glomerular extracellular proteins *in situ* yields more accurate results.

Applying the assay in an acute and a chronic proteinuric disease model, we demonstrate that the amount of surface nephrin can be reliably quantified with the biotinylation assay introduced.

## Materials and methods

### Animal care

Mice were obtained either from an in-house breed at a local animal care facility or from Janvier Labs, France. This study was carried out in strict accordance with the recommendations in the Guide for the Care and Use of Laboratory Animals of the National Institutes of Health. The protocol was approved by the local Institutional Animal Care and Use Committee: Landesamt für Natur, Umwelt und Verbraucherschutz Nordrhein-Westfalen, Recklinghausen, Germany; Permit Number: AZ: 84–02.04.2012.A099. If necessary analgesia was performed with Carprofen 5 mg/kg s.c. Animals were sacrificed by decapitation under anesthesia performed with Ketamin 100mg/kg bodyweight and Xylazin 5mg/kg bodyweight.

### Induction of nephrotoxic serum nephritis (NTN)

C57Bl/6 male mice (8 weeks of age) were injected intraperitoneally with 500–800 μL NTN serum. Control animals received an equal amount of normal saline. Following the collection of urine, mice were weighed and examined on day 1 or 18 after injection.

### Induction of adriamycin-induced nephropathy (ADR)

BALB/c male mice (6 weeks of age) were injected intravenously with adriamycin (10 mg/kg bodyweight; ADR, Sigma-Aldrich, Steinheim, Germany), and control animals were administered an equivalent volume of water for injection (aqua ad injectabilia). Following the collection of urine, mice were weighed and examined 7 days after injection.

### Analysis of proteinuria

Twelve hour urine collection was performed in single metabolic cages. Proteinuria was assessed using a 10% polyacrylamide gel. Urinary albumin and creatinine excretion were measured using standard laboratory protocols, and proteinuria was normalized based on creatinine excretion.

### *In vivo* biotin labeling and immunoprecipitation (IP)

First, kidneys were perfused via the abdominal aorta with 5ml ice-cold PBS (rate 2mL/min) supplemented with 1 mM MgCl_2_ and 0.1 mM CaCl_2_ (PBSCM). Then, perfusion was repeated with 5 mL PBSCM (rate 2mL/min) supplemented with 0.5 mg/mL EZ-Link^™^ Sulfo-NHS-LC-Biotin (Thermo Scientific, Rockford, USA) for surface protein labeling. Afterwards, unspecific biotin binding was quenched with 5mL PBSCM (rate 2mL/min) supplemented with 100 mM glycine. Finally, kidneys were perfused with 5 mL PBSCM (rate 2mL/min) containing 16 x 10^6^ Dynabeads/mL (Invitrogen, Oslo, Norway). After perfusion, kidneys were immediately minced and digested for 40 minutes at 37°C with 1.5 mg/mL Collagenase A (Roche, Mannheim, Germany). Cell suspensions were filtered through 100 μm cell strainers (Greiner Bio-One, Frickenhausen, Germany), separated by centrifugation (5.000 ×*g* for 5 min at 4°C), and washed with PBSCM using a Dynamag magnet. When a purity of >95% glomeruli was achieved, glomeruli were collected by centrifugation (6.800 ×*g* for 5 min at 4°C). Samples were immediately homogenized using a TissueRuptor (Qiagen, Hombrechtikon, Switzerland), and were lysed for 30 min on ice. Insoluble cellular material was removed by centrifugation (15.000 ×*g* for 30 min at 4°C). Protein concentrations from the supernatant were measured using a BCA Protein assay kit (Thermo Scientific, Rockford, USA), and were subsequently adjusted to ensure equal total protein content.

IP analysis: samples were incubated with anti-nephrin antibodies overnight at 4°C, followed by a 3-h incubation with protein A-Sepharose (GE LifeSciences, Freiburg, Germany) or with or streptavidin agarose beads directly (Pierce, Thermo Fisher Scientific, Waltham, USA). Due to N-linked glycosylation nephrin appears as a double band [17]. The immunoprecipitates were extensively washed with CHAPS buffer. Bound proteins were resolved using 2× Laemmli sample buffer, separated on 10% polyacrylamide gel, and electroblotted onto nitrocellulose membranes. The blots were blocked in 5% bovine serum albumin (BSA) in TBST before incubation with primary antibodies (nephrin: Progen GP-N2, Heidelberg, Germany; beta-actin: Sigma, St. Louis, USA; streptavidin-HRP: Thermo Scientific, Rockford, USA; p44/42 MAPK (Erk1/2): Cell Signaling Technologies, Danvers, USA; Podocalyxin: R&D Systems, Minneapolis, USA) overnight at 4°C. After washing with TBST for 30 min, the blots were incubated for 60 min with HRP-coupled secondary antibodies, and excessive antibodies were removed by washing with TBST for 30 min. ECL SuperSignal (Thermo Scientific, Rockford, U.S.A.) was used for chemiluminescence visualization (FluorChem FC2 Imager; Alpha Innotec, USA), and the densitometric analysis was performed (AlphaView SA; Cell Biosciences Inc., version 3.3.1, Alpha Innotec, USA).

### Immunofluorescence

#### Biotin—Streptavidin

For immunofluorescent stainings, 2 μm paraffin sections were deparaffinized and antigen retrieval was performed by boiling at 98°C in 0.05% citraconic acid anhydride (Sigma, St. Louis, USA), pH7.4 for 20 min. Unspecific binding was blocked in 3% goat serum for 60 min at RT. Primary antibody incubation (rabbit α-WT1 1:800, Santa-Cruz, Santa-Cruz, USA) was performed in blocking buffer o/n at 4°C. Binding was visualized by incubation with TRITC-α-rabbit coupled secondary antibodies (Abcam, Eugene, USA) diluted 1:300 in blocking buffer for 60 min at RT. Stainings were evaluated with a Zeiss Axio Observer microscope using the LSM software (Zeiss, Jena, Germany).

#### Nephrin—EEA1

For immunofluorescent stainings, 2 μm paraffin sections were deparaffinized and antigen retrieval was performed by boiling at 98°C in 0.05% citraconic acid anhydride (Sigma, St. Louis, USA), pH7.4 for 40 min. Unspecific binding was blocked in 5% horse serum with 0.05% Triton X-100 for 30 min at RT. Primary antibody incubations (guinea-pig α-nephrin (IF 1:200, Acris, Rockville, USA) and rabbit α-EEA1 (IF 1:400, Santa Cruz, Santa Cruz, USA) were performed in blocking buffer o/n at 4°C. Binding was visualized by incubation with AF488-α-guinea pig or Cy3-α-rabbit coupled secondary antibodies (all affinity-purified donkey antibodies Jackson ImmunoResearch, West Grove, USA) diluted 1:400 in blocking buffer for 30 min at RT. Stainings were evaluated with a Zeiss LSM 510 meta microscope using the LSM software (all Zeiss, Jena, Germany).

### Quantification of podocyte loss

As described before staining of the CDK-inhibitor p57 was used to measure the number of healthy podocytes [18, 19]. Two μm thick paraffin sections were deparaffinized and antigen retrieval was performed by boiling for 30 min in 10 mM citrate buffer pH 6.1 at constant 98°C in a steam cooker. Unspecific binding was blocked (5% horse serum, 30 min RT). Rabbit anti-p57 (Santa Cruz, Santa Cruz, USA) diluted 1:400 in 5% horse serum was incubated o/n at 4°C. Color development was performed with the ZytoChem-Plus AP Polymer-Kit (Zytomed, Berlin, Germany) according to the manufacturers’ instructions with neufuchsin. Stainings were evaluated under an Axioskop and photographed with an Axiocam HRc using the Axiostar software (all Zeiss, Jena, Germany). Quantification of podocyte loss was performed using a serpentine movement from cortex to medulla and vice versa. The outlines and p57-positive nuclei of 40 consecutively encountered capillary tufts were traced and counted manually at a 400-fold magnification in a blinded manner. The mean podocyte number per glomerular random cross-sectional area was calculated.

## Results

### Quantification of nephrin surface expression in mice

**Fig 1:**
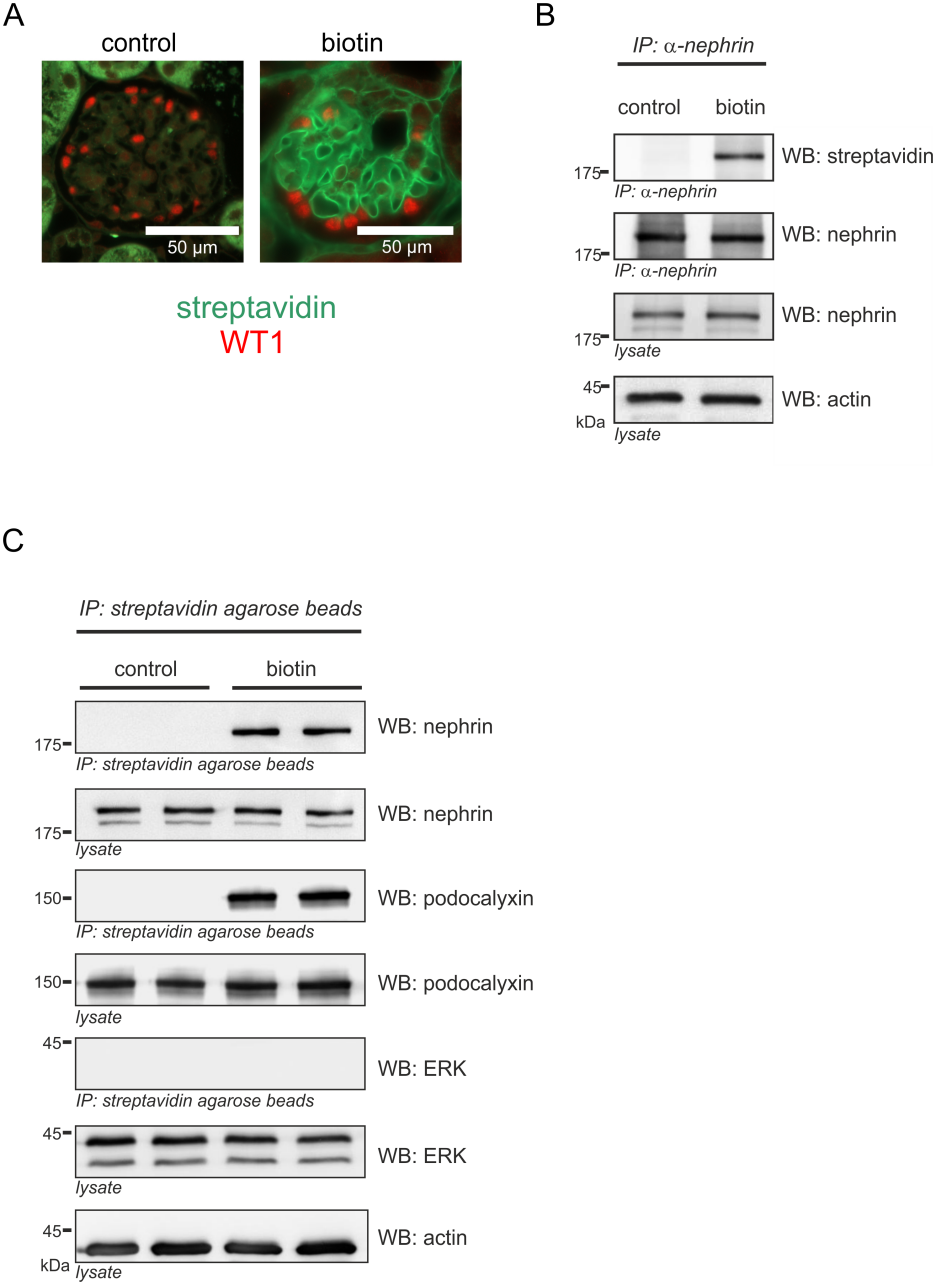
Detection of biotin in murine glomeruli. **(A)** Representative immunofluorescence staining of kidney sections of C57Bl/6 mice. Podocyte nuclei were labeled by WT1 staining (red). Biotin was detected with streptavidin (green). In biotin-perfused mice (biotin), biotin was detected along the glomerular capillaries. PBS-perfused mice (control) did not show any biotin deposition. **(B)** Western blot analysis after immunoprecipitation of nephrin (IP α-nephrin) from biotin-perfused (biotin) and PBS-perfused (control) murine kidneys. A biotinylated fraction of nephrin (WB streptavidin) was only detectable in biotin-perfused mice. Total nephrin immunoprecipitates and lysates (WB nephrin) showed equal expression of total nephrin. Beta-actin (actin) was used as a loading control. **(C)** Western blot analysis after immunoprecipitation of biotinylated surface proteins (IP streptavidin agarose beads) from biotin-perfused (biotin) and PBS-perfused (control) murine kidneys. Biotinylated nephrin (WB nephrin) and podocalyxin (WB podocalyxin) were only detectable in biotin-perfused mice. Extracellular-signal regulated kinases p42 and p44 (ERK) could not be detected at all. Total nephrin, podocalyxin and ERK lysates (WB nephrin, WB podocalyxin and WB ERK) showed equal expression of nephrin, podocalyxin and ERK. Beta-actin (actin) was used as a loading control.

Mice were perfused with either biotin or phosphate buffered saline (PBS). In kidneys perfused with biotin, linear staining along the glomerular capillaries was visualized. No staining was detected in controls perfused with PBS (Fig 1A). To quantify the surface fraction of nephrin two different approaches have been used. First, nephrin was separated by immunoprecipitation with a nephrin specific antibody, followed by SDS-PAGE and western blot analyses. The biotinylated fraction of nephrin, was then visualized by staining with streptavidin. No biotinylated nephrin was detected in PBS perfused controls. Lysate controls indicated equal amounts of protein (Fig 1B). Second, the reverse experiment has been performed. Biotinylated proteins were separated by pull-down with strepatividin agarose beads, followed by SDS-PAGE and western blot analyses. The nephrin fraction was visualized by staining with a nephrin specific antibody. To demonstrate that the assay allows the detection of other glomerular surface proteins podocalyxin was detected. The intracellular control proteins extracellular-signal regulated kinases p42 and p44 (ERK) could not be detected. Further, no biotinylated proteins were detected in PBS perfused controls. Lysate controls indicated equal amounts of protein. (Fig 1C).

### Nephrin surface expression in nephrotoxic nephritis

#### Early nephrotoxic nephritis

**Fig 2:**
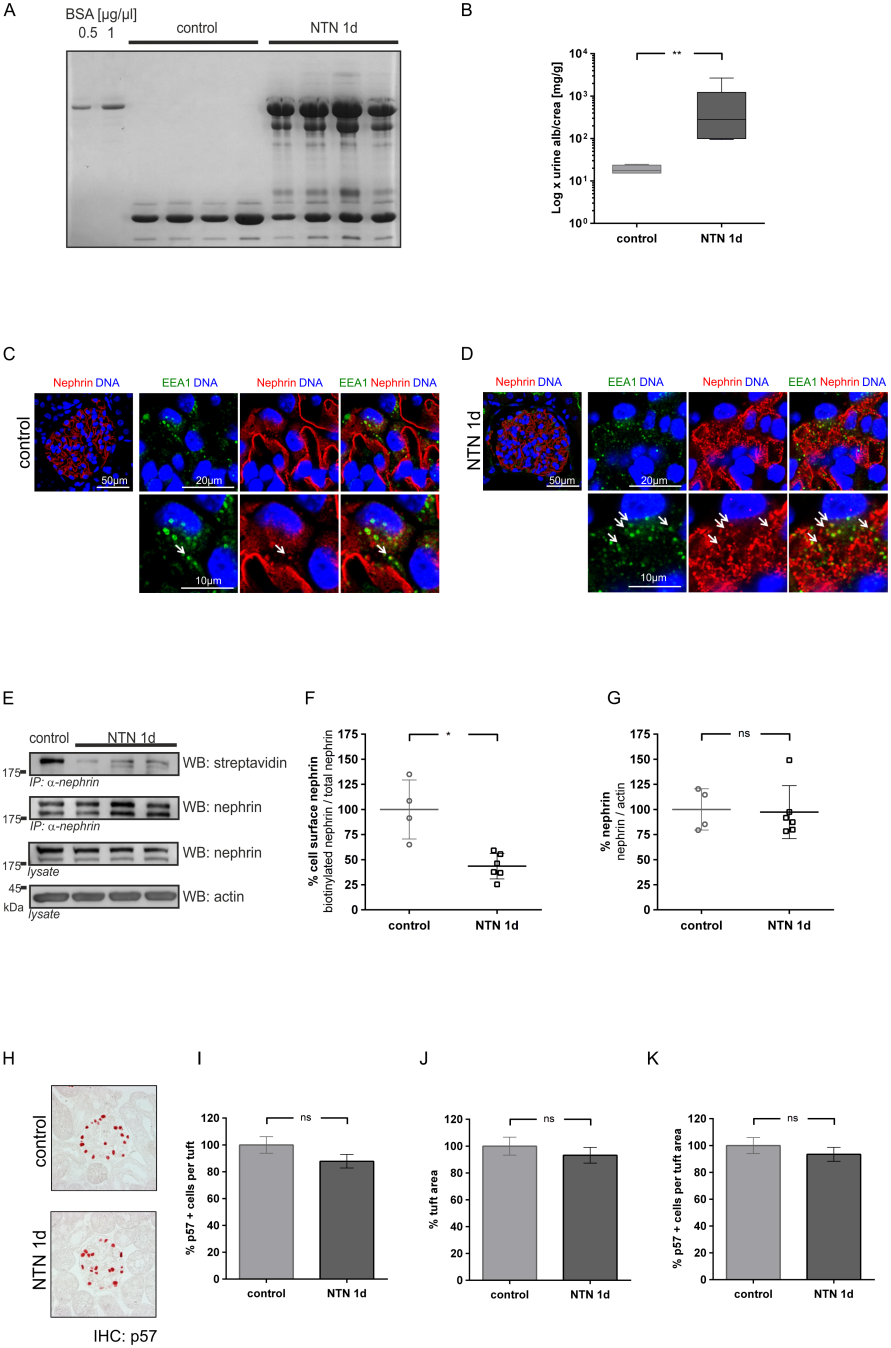
Albuminuria and nephrin surface abundance in early nephrotoxic nephritis. **(A)** Coomassie gel shows albuminuria in mice injected with NTN serum (NTN 1d). No albuminuria was detected in mice injected with normal saline (control). **(B)** Quantitative evaluation of albumin-creatinine excretion in healthy mice (control) and NTN mice (NTN 1d). Statistical analysis: Mann-Whitney U test, ** p < 0.01 (control: n = 4; NTN: n = 6). **(C, D)** Immunofluorescence staining of murine kidney sections of healthy mice (control) and mice injected with NTN serum (NTN 1d). Staining was performed with an anti-nephrin antibody (red), an anti-EEA1-antibody (green) and nuclear DNA with Draq5 (blue). White arrows indicate colocalization of nephrin with EEA1 positive vesicles. **(E)** Representative western blot of total and biotinylated nephrin detected with streptavidin (streptavidin). In comparison to untreated mice (control) less surface nephrin (WB: streptavidin) was detected in NTN mice (NTN 1 d). Total nephrin immunoprecipitates and lysates (WB: nephrin) showed equal expression of total nephrin in both groups. β-actin (WB: actin) was used as loading control. **(F, G)** Densitometric analysis of western blots: **(F)** Amount of nephrin at the cell surface graphed as biotinylated nephrin to total nephrin ratio; **(G)** Amount of total nephrin graphed as total nephrin to β-actin ratio. **(H)** Immunohistochemistry: staining of p57 indicates podocytes in healthy mice (control) and NTN mice (NTN d1). **(I)** Number of p57 positive cells per glomerular tuft **(J)** Glomerular tuft area (μm^2^) **(K)** Percentage of p57 positive cells in relation to tuft area. (40 gloms per mouse quantified). Western Blot data show means ± SD. Podocyte counts show means ± SEM. Statistical analysis: unpaired *t*-test with Welch’s correction. Significance level was set to 5%, *p < 0.01, ** p<0.001, (non-significant differences (ns)).

Nephrotoxic nephritis (NTN) rapidly induces severe proteinuria in its heterologous phase [20]. One day after injection of the NTN serum, proteinuria in mice significantly increased 3–4 log ranks (Fig 2A and 2B). In comparison to untreated kidneys induction of NTN led to a transformation of the linear staining of nephrin to a punctate pattern. High resolution images show an enhanced colocalization of nephrin with early endosomal antigen 1 (EEA-1) (Fig 2C and 2D), suggesting increased endocytosis of the nephrin molecules. Loss of nephrin from the slit diaphragm was then quantified using the biotinylation assay (Fig 2E). At day 1, densitometric analysis of the western blot results indicated a 57% decrease of biotinylated nephrin in NTN mice (Fig 2F), but equal amounts of total nephrin were detected (Fig 2G). In line with this finding the podocyte numbers remained stable in the NTN mice compared to untreated animals (Fig 2H–2K).

#### Late nephrotoxic nephritis

**Fig 3:**
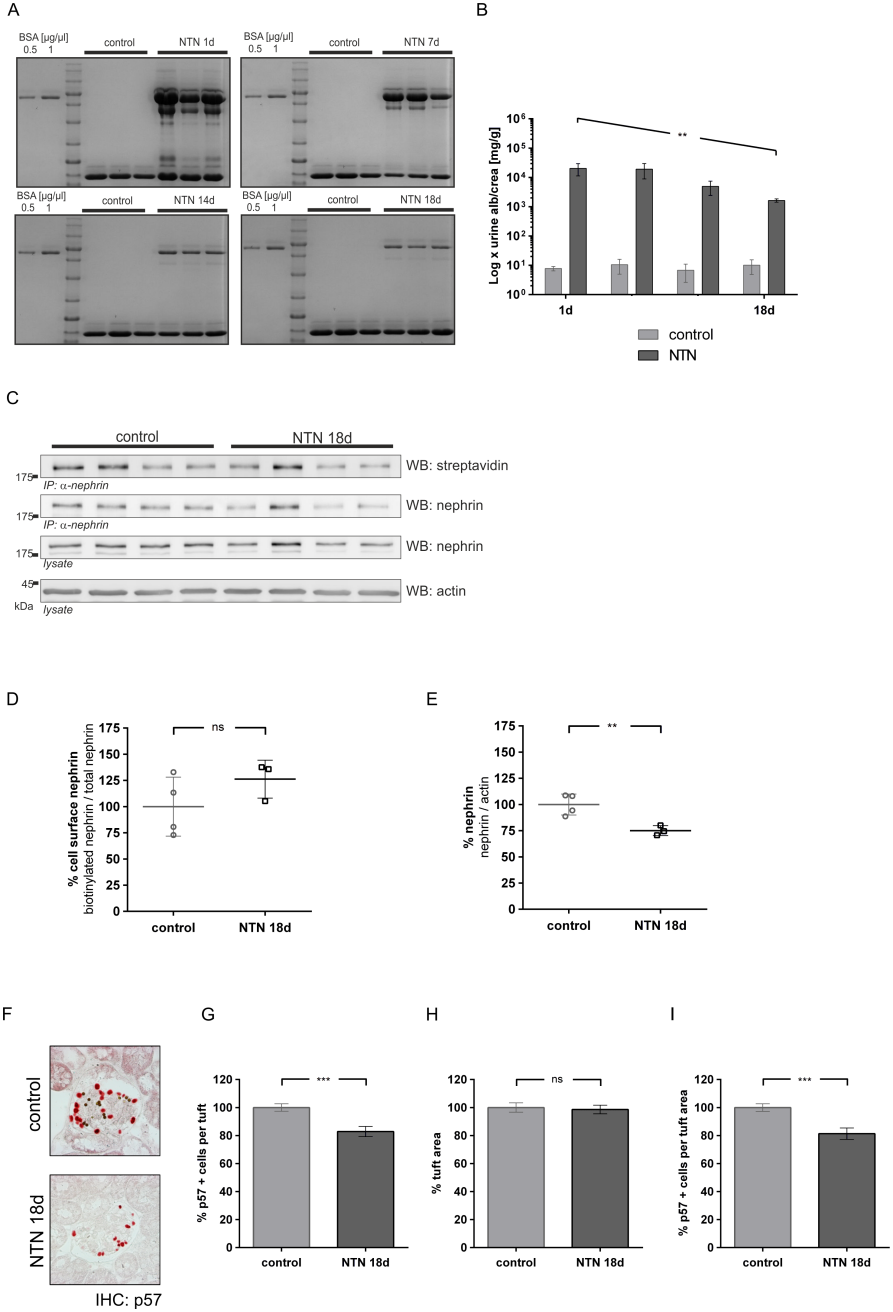
Albuminuria and nephrin surface abundance in late nephrotoxic nephritis. **(A)** Coomassie gel loaded with urine samples collected on days 1, 7, 14, and 18 after serum injection (BSA standard, left). In NTN mice, albuminuria decreased over the course of 18 days. **(B)** Quantitative analysis of albumin to creatinine excretion in healthy (light grey) and NTN mice (dark grey) on days 1, 7, 14, and 18. Statistical analysis: one-way ANOVA test for linear trend, ** p < 0.005 (control: n = 4; NTN: n = 3). **(C)** Western blot analysis of total nephrin and biotinylated nephrin (streptavidin) from control mice (control) versus NTN mice 18 days (NTN 18 d) after serum injection. In comparison to control mice (control), less surface nephrin (WB: streptavidin) was detected in NTN mice (NTN 18 d). Total nephrin immunoprecipitates and lysates (WB: nephrin) demonstrated less expression of total nephrin after 18 days. β-actin (WB: actin) was used as the loading control. **(D, E)** Densitometric analysis of western blots: **(D)** Amount of surface nephrin graphed as biotinylated nephrin to total nephrin ratio; **(E)** Amount of total nephrin graphed as total nephrin to β-actin ratio. **(F)** Immunohistochemistry: staining of p57 (red) indicates podocytes in healthy mice (control) and NTN mice (NTN 1d). Magnetic beads appear as black dots. **(G)** Number of p57 positive cells per glomerular tuft **(H)** Glomerular tuft area (μm^2^) **(I)** Percentage of p57 positive cells in relation to tuft area (control n = 2, NTN n = 2, 40 gloms per mouse quantified). Western blot data show means ± SD. Podocyte counts show means ± SEM. Statistical analysis: unpaired *t*-test with Welch’s correction. Significance level was set to 5%, ** p < 0.001, *** p<0.0001, (non-significant differences (ns)).

Albuminuria decreased significantly over the course of 18 days (Fig 3A and 3B). At day 18, the results of the western blot analysis conducted after biotinylation indicated the recovery of surface nephrin (Fig 3C). At day 18, the densitometric results showed no significant differences between the percentage of cell surface nephrin in either group (Fig 3D). However, total nephrin was reduced by 25% in NTN mice at day 18 (Fig 3E). Podocyte numbers were reduced by approximately 19% in NTN mice (Fig 3F–3J).

### Adriamycin induced nephropathy

**Fig 4:**
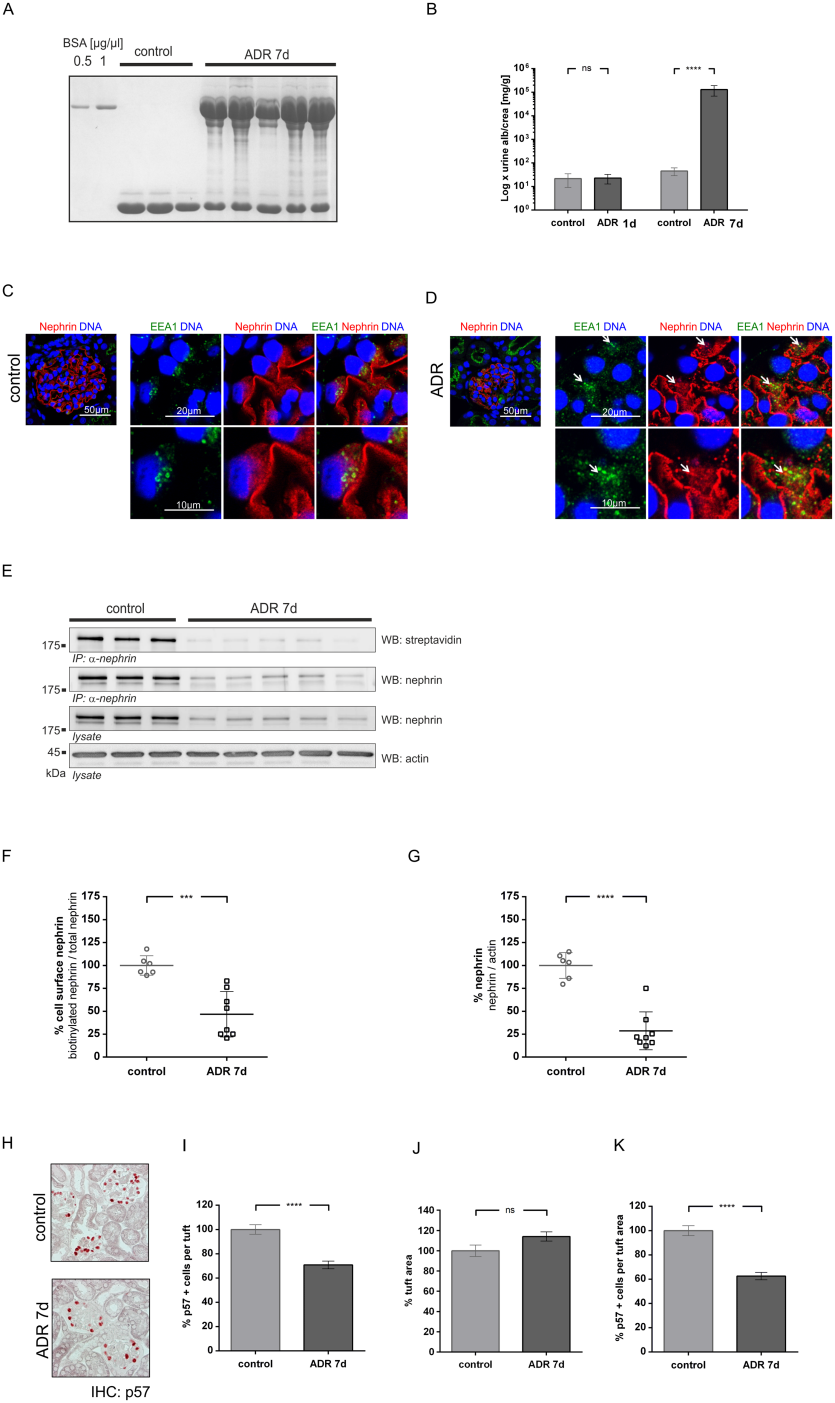
Nephrin surface abundance in adriamycin nephropathy at day 7. **(A)** Coomassie gel shows albuminuria in ADR mice at day 7 (ADR 7d), which was not detected in healthy mice (control). **(B)** Quantitative analysis of the albumin to creatinine ratio in healthy mice (control) versus ADR mice on days 1 and 7 (ADR1 and ADR 7). Statistical analysis: *t*-test with Welch’s correction was performed on day 1 and 7. **** p < 0.0001 (non-significant differences (ns); control: n = 9; ADR: n = 12). **(C, D)** Immunofluorescence staining of glomeruli from healthy mice (control) or ADR mice (ADR 7 d). Staining was performed with an anti-nephrin antibody (red), and nuclei were stained with DAPI (blue). White arrows indicate colocalization of nephrin with EEA1 positive vesicles. **(E)** Western blot analysis of surface nephrin (streptavidin) and total nephrin immunoprecipitates and lysates (WB nephrin). In comparison to healthy mice (control), less surface nephrin (WB: streptavidin) was detected in ADR mice at day 7 (ADR 7d). Total nephrin immunoprecipitates and lysates (WB: nephrin) showed less expression of total nephrin at day 7. β-actin (WB: actin) was used as loading control. **(F, G)** Densitometric analysis of western blots: **(F)** Amount of cell surface nephrin graphed as biotinylated nephrin to total nephrin ratio; **(G)** Amount of total nephrin graphed as total nephrin to β-actin ratio. **(H)** Immunohistochemistry: staining of p57 indicates podocytes in healthy mice (control) and NTN mice (NTN d1). **(I)** Number of p57 positive cells per glomerular tuft **(J)** Glomerular tuft area (μm^2^) **(K)** Ratio of p57 positive cells in relation to tuft area (control n = 2, ADR n = 3, 40 gloms per mouse quantified). Western blot data show means ± SD. Podocyte counts show means ± SEM. Statistical analysis: unpaired *t*-test with Welch’s correction. Significance level was set to 5%, *** p < 0.0001, **** p<0.00001, (non-significant differences (ns)).

Adriamycin induced nephropathy (ADR) is a model of focal segmental sclerosis [21]. Six days after injection, a significant four log rank increase of proteinuria (compared to controls) was detected (Fig 4A and 4B). In comparison to healthy mice adriamycin induced a disruption of the linear staining pattern of nephrin, which was replaced by a fragmented and granulated cytoplasmic staining pattern. High resolution images show an enhanced colocalization of nephrin with EEA-1, suggesting endocytosis of nephrin (Fig 4C and 4D). These findings were confirmed in the *in vivo* biotinylation assay that was conducted using a western blot analysis (Fig 4E). Densitometric evaluation of the results at day 7 revealed that, despite a reduction of total nephrin by 71% (Fig 4F), biotinylated nephrin was still significantly decreased by 53% (Fig 4G). Podocyte numbers were reduced by approximately 40% (Fig 4H–4K).

## Discussion

The *in vivo* biotinylation assay allowed the quantification of nephrin abundance at the glomerular slit diaphragm of mice. This method is useful as a complementary technique to immunofluorescence, and expands the possibilities of studies focusing on the expression and trafficking of glomerular surface proteins. As proof of principle the hallmark proteins of the podocyte nephrin and podocalyxin have been detected. To demonstrate the usefulness of the assay, we quantified nephrin surface abundance in two different and well-established models of proteinuric kidney disease.

NTN led to the rapid induction of albuminuria, which was nearly reversible in the course of disease. Quantification of nephrin revealed that more than half of the molecules were lost from the slit diaphragm at the peak of albuminuria, while the amount of total nephrin was preserved. In addition, the results of immunofluorescence studies showed a change of the linear distribution of nephrin to a punctate pattern. Colocalization experiments revealed that nephrin was translocated from the podocyte surface to the early endosome suggesting endocytosis of nephrin. Besides endocytosis changes in podocyte morphology and loss of podocytes presumably contribute to the observed pattern change. These findings are in line with previous results [8]. When albuminuria decreased once again, the surface abundance of nephrin was restored. However, after 18 days, nephrin abundance was diminished by approximately 25%. Histologic analysis of podocyte numbers suggested that this reduction in total nephrin was most likely caused by loss of podocytes. Our finding is also in line with previous reports of nephrotoxic nephritis [22]. Regarding ADR, albuminuric animals also showed significant reductions of surface nephrin. Immunofluorescence revealed an enhanced translocation of nephrin in vesicles of the early endosome. Moreover, there was a marked reduction of total nephrin most likely due to the loss of podocytes. This finding has been reported for ADR earlier [23], and is supported by our observation that podocyte numbers are strongly reduced in ADR at day 7. It should be kept in mind that an impaired perfusion with the magnetic particles due to advanced damage of the glomerular tuft could lead to a biased quantification of glomerular proteins. However, it might be questioned whether the inside of damaged capillary tufts would be adequately covered with biotin when extracted glomeruli are bathed in biotin like described by Satoh et al. [16]. To account for fluctuations in total protein yield and to ensure accurate quantitation and comparability between different experiments, all Western blot replicates have been normalized to beta actin.

## Conclusion

In both models of proteinuric kidney disease nephrin surface abundance correlated inversely with proteinuria. In nephrotoxic nephritis we could show that proteinuria and nephrin loss were reversible. Taken together this proof-of-concept study shows the potential of the *in vivo* biotinylation assay to gain more insight into the function of the glomerular filter.

## References

[1] Tsai WC, Wu HY, Peng YS, Ko MJ, Wu MS, Hung KY, et al Risk Factors for Development and Progression of Chronic Kidney Disease: A Systematic Review and Exploratory Meta-Analysis. Medicine. 2016;95(11):e3013 Epub 2016/03/18. doi: 10.1097/MD.0000000000003013 ; 26986114 10.1097/MD.0000000000003013 PMC4839895

[2] Kalaitzidis R, Bakris G. Pathogenesis and treatment of microalbuminuria in patients with diabetes: the road ahead. J Clin Hypertens (Greenwich). 2009;11(11):636–43. Epub 2009/11/03. doi: 10.1111/j.1751-7176.2009.00184.x . 19878372 10.1111/j.1751-7176.2009.00184.x PMC8673001

[3] Quack I, Rump LC, Gerke P, Walther I, Vinke T, Vonend O, et al beta-Arrestin2 mediates nephrin endocytosis and impairs slit diaphragm integrity. Proceedings of the National Academy of Sciences of the United States of America. 2006;103(38):14110–5. Epub 2006/09/14. doi: 10.1073/pnas.0602587103 ; 16968782 10.1073/pnas.0602587103 PMC1564064

[4] Quack I, Woznowski M, Potthoff SA, Palmer R, Konigshausen E, Sivritas S, et al PKC alpha mediates beta-arrestin2-dependent nephrin endocytosis in hyperglycemia. The Journal of biological chemistry. 2011;286(15):12959–70. Epub 2011/02/16. doi: 10.1074/jbc.M110.204024 ; 21321125 10.1074/jbc.M110.204024 PMC3075643

[5] Inoue K, Ishibe S. Podocyte endocytosis in the regulation of the glomerular filtration barrier. American journal of physiology Renal physiology. 2015;309(5):F398–405. Epub 2015/06/19. doi: 10.1152/ajprenal.00136.2015 ; 26084928 10.1152/ajprenal.00136.2015 PMC4556893

[6] Soda K, Ishibe S. The function of endocytosis in podocytes. Current opinion in nephrology and hypertension. 2013;22(4):432–8. Epub 2013/05/25. doi: 10.1097/MNH.0b013e3283624820 ; 23703394 10.1097/MNH.0b013e3283624820 PMC4143890

[7] Swiatecka-Urban A. Endocytic Trafficking at the Mature Podocyte Slit Diaphragm. Front Pediatr. 2017;5:32 doi: 10.3389/fped.2017.00032 ; 28286744 10.3389/fped.2017.00032 PMC5324021

[8] Babayeva S, Rocque B, Aoudjit L, Zilber Y, Li J, Baldwin C, et al Planar cell polarity pathway regulates nephrin endocytosis in developing podocytes. The Journal of biological chemistry. 2013;288(33):24035–48. Epub 2013/07/05. doi: 10.1074/jbc.M113.452904 ; 23824190 10.1074/jbc.M113.452904 PMC3745348

[9] Babayeva S, Zilber Y, Torban E. Planar cell polarity pathway regulates actin rearrangement, cell shape, motility, and nephrin distribution in podocytes. American journal of physiology Renal physiology. 2011;300(2):F549–60. Epub 2010/06/11. doi: 10.1152/ajprenal.00566.2009 . 20534871 10.1152/ajprenal.00566.2009

[10] Teng B, Schroder P, Muller-Deile J, Schenk H, Staggs L, Tossidou I, et al CIN85 deficiency prevents nephrin endocytosis and proteinuria in diabetes. Diabetes. 2016 Epub 2016/08/18. doi: 10.2337/db16-0081 . 27531950 10.2337/db16-0081 PMC5314701

[11] Tossidou I, Teng B, Drobot L, Meyer-Schwesinger C, Worthmann K, Haller H, et al CIN85/RukL is a novel binding partner of nephrin and podocin and mediates slit diaphragm turnover in podocytes. The Journal of biological chemistry. 2010;285(33):25285–95. Epub 2010/05/12. doi: 10.1074/jbc.M109.087239 ; 20457601 10.1074/jbc.M109.087239 PMC2919091

[12] Waters AM, Wu MY, Huang YW, Liu GY, Holmyard D, Onay T, et al Notch promotes dynamin-dependent endocytosis of nephrin. Journal of the American Society of Nephrology: JASN. 2012;23(1):27–35. Epub 2011/11/05. doi: 10.1681/ASN.2011010027 ; 22052054 10.1681/ASN.2011010027 PMC3269920

[13] Qin XS, Tsukaguchi H, Shono A, Yamamoto A, Kurihara H, Doi T. Phosphorylation of nephrin triggers its internalization by raft-mediated endocytosis. Journal of the American Society of Nephrology: JASN. 2009;20(12):2534–45. Epub 2009/10/24. doi: 10.1681/ASN.2009010011 ; 19850954 10.1681/ASN.2009010011 PMC2794235

[14] Soda K, Balkin DM, Ferguson SM, Paradise S, Milosevic I, Giovedi S, et al Role of dynamin, synaptojanin, and endophilin in podocyte foot processes. The Journal of clinical investigation. 2012;122(12):4401–11. Epub 2012/11/29. doi: 10.1172/JCI65289 ; 23187129 10.1172/JCI65289 PMC3533561

[15] Dundas CM, Demonte D, Park S. Streptavidin-biotin technology: improvements and innovations in chemical and biological applications. Applied microbiology and biotechnology. 2013;97(21):9343–53. Epub 2013/09/24. doi: 10.1007/s00253-013-5232-z . 24057405 10.1007/s00253-013-5232-z

[16] Satoh D, Hirose T, Harita Y, Daimon C, Harada T, Kurihara H, et al aPKClambda maintains the integrity of the glomerular slit diaphragm through trafficking of nephrin to the cell surface. J Biochem. 2014;156(2):115–28. doi: 10.1093/jb/mvu022 ; 24700503 10.1093/jb/mvu022 PMC4112437

[17] Yan K, Khoshnoodi J, Ruotsalainen V, Tryggvason K. N-linked glycosylation is critical for the plasma membrane localization of nephrin. Journal of the American Society of Nephrology: JASN. 2002;13(5):1385–9. . 11961028 10.1097/01.asn.0000013297.11876.5b

[18] Hiromura K, Haseley LA, Zhang P, Monkawa T, Durvasula R, Petermann AT, et al Podocyte expression of the CDK-inhibitor p57 during development and disease. Kidney Int. 2001;60(6):2235–46. doi: 10.1046/j.1523-1755.2001.00057.x . 11737597 10.1046/j.1523-1755.2001.00057.x

[19] Taniguchi Y, Pippin JW, Hagmann H, Krofft RD, Chang AM, Zhang J, et al Both cyclin I and p35 are required for maximal survival benefit of cyclin-dependent kinase 5 in kidney podocytes. American journal of physiology Renal physiology. 2012;302(9):F1161–71. doi: 10.1152/ajprenal.00614.2011 ; 22262481 10.1152/ajprenal.00614.2011 PMC3362174

[20] Assmann KJ, Tangelder MM, Lange WP, Schrijver G, Koene RA. Anti-GBM nephritis in the mouse: severe proteinuria in the heterologous phase. Virchows Archiv A, Pathological anatomy and histopathology. 1985;406(3):285–99. Epub 1985/01/01. . 3923705 10.1007/BF00704298

[21] Lee VW, Harris DC. Adriamycin nephropathy: a model of focal segmental glomerulosclerosis. Nephrology (Carlton). 2011;16(1):30–8. Epub 2010/12/24. doi: 10.1111/j.1440-1797.2010.01383.x . 21175974 10.1111/j.1440-1797.2010.01383.x

[22] Sussman AN, Sun T, Krofft RM, Durvasula RV. SPARC accelerates disease progression in experimental crescentic glomerulonephritis. The American journal of pathology. 2009;174(5):1827–36. Epub 2009/04/04. doi: 10.2353/ajpath.2009.080464 ; 19342370 10.2353/ajpath.2009.080464 PMC2671271

[23] Pereira Wde F, Brito-Melo GE, de Almeida CA, Moreira LL, Cordeiro CW, Carvalho TG, et al The experimental model of nephrotic syndrome induced by Doxorubicin in rodents: an update. Inflammation research: official journal of the European Histamine Research Society [et al]. 2015;64(5):287–301. Epub 2015/03/20. doi: 10.1007/s00011-015-0813-1 . 25788426 10.1007/s00011-015-0813-1

